# Effect of Fat-Soluble Anti-oxidants in Vegetable Oils on Acrylamide Concentrations During Deep-Fat Frying of French Fries

**DOI:** 10.21315/mjms2018.25.5.12

**Published:** 2018-10-30

**Authors:** Siti Asiah Kamarudin, Selamat Jinap, Rashidah Sukor, Siew Pheng Foo, Maimunah Sanny

**Affiliations:** 1 Department of Food Science, Faculty of Food Science and Technology, Universiti Putra Malaysia, 43400 Serdang, Selangor, Malaysia; 2 Laboratory of Food Safety and Food Integrity (FOSFI), Institute of Tropical Agriculture and Food Security, Universiti Putra Malaysia, 43400 UPM, Serdang, Selangor, Malaysia; 3 Carotino Sdn. Bhd., PLO 519 Jalan Besi Satu, Pasir Gudang Industrial Estate, 81700 Pasir Gudang, Johor, Malaysia

**Keywords:** fat-soluble anti-oxidants, vegetable oils, acrylamide, French fries

## Abstract

**Background:**

This study attempted to evaluate the effect of fat-soluble anti-oxidants in vegetable oils on acrylamide during the deep-fat frying of French fries.

**Methods:**

Three vegetable oils with different fat-soluble anti-oxidant contents were selected and par-fried potato strips were fried in these oils. Acrylamide in the French fries at different frying times (at 180 °C) and over 10 consecutive frying sessions were measured. The anti-oxidant contents and quality degradation of oils were monitored before and after the 5th and 10th consecutive frying sessions.

**Results:**

The effect of the fat-soluble anti-oxidants in red palm oil on the acrylamide was more apparent when a prolonged frying time was used for consecutive frying sessions than when different frying conditions were used. Using red palm oil, acrylamide concentration in French fries significantly dropped to the lowest level, at 524 ng g^−1^, after the 10th frying session. The β-carotene content after the 10th frying session was the highest in red palm oil.

**Conclusion:**

The use of red palm oil for deep-fat frying French fries can be a mitigation strategy to reduce acrylamide formation, but further studies are necessary to investigate the influence of different types of fat-soluble anti-oxidants on the inhibition of acrylamide formation.

## Introduction

Acrylamide, a probable carcinogen was discovered by Swedish National Food Authority in 2002 in a variety of baked and fried foods cooked at high temperature. Considerable health concerns have been raised world-wide due to the reports (1). International Agency for Research on Cancer (IARC) classified acrylamide as a potential carcinogen to human (Group 2A) based on its carcinogenicity in rodents (2). Acrylamide has been found to increase incidences of benign and malignant tumours in organ (thyroid and adrenal) (3). High intake of acrylamide could also increase the risk of kidney and breast cancer (4, 5). Plant-based foods that are dense in carbohydrate such as potatoes, cereals and many more served as a source of formation of acrylamide. The Maillard reaction is the main mechanistic pathway established for acrylamide formation, and free asparagine is the main precursor (6). French fries are a potato-based product that contains high concentrations of acrylamide (7) and its ‘benchmark value’ recently been established lower (500 ng g^−1^) than the ‘indicative value’ (600 ng g^−1^). Should the acrylamide levels surpass the prescribed benchmark values, investigations are recommended (8).

Numerous researchers have investigated various mitigation strategies to minimise the acrylamide concentrations in processed foods (9, 10). Different authors have used anti-oxidants to minimise acrylamide formation (11, 12). For example, the addition of vitamin E (0.1 mg kg^−1^) to a cookie formula was reported to mitigate the formation of acrylamide by 49.6% (11). Further, water-soluble anti-oxidants in virgin olive oil reduce acrylamide formation in crisps under different frying conditions (12). Nonetheless, Tareke (13) reported that the addition of vitamin E to meat before heating enhances the formation of acrylamide. Jin et al. (14) explained that the previously mentioned discordant effects may occur due to the ability of anti-oxidants with different structures that reacted with acrylamide precursors, with intermediates of the reaction or with acrylamide itself, heading to either reducing or promoting effects. Further, Constantinou and Koutsidis (15) discussed that the discordant effects may also be attributed to the matrix effects, the anti-oxidant type and the anti-oxidant content.

During frying, food is immersed in a hot oil at a high temperature of 150 °C to 190 °C (16). Deep frying is often performed using vegetable oil that contains one or more of several oils, such as corn, canola, soybean, cottonseed, sunflower, and palm oil (17). Tocopherols and tocotrienols are the major fat-soluble anti-oxidants in vegetable oils (18). Dauqan et al. (19) reported that the content and the percent composition of tocopherols and tocotrienols were higher in palm oil and red palm oil compared to corn oil. The β-carotene content is the highest in red palm oil and the lowest in corn oil (19). Napolitano et al. (12) suggested that the anti-oxidant properties of vegetable oils from different sources have to be considered when analysing acrylamide formation. Furthermore, there are limited studies on the effect of fat-soluble anti-oxidants on the formation of acrylamide. The research to date has focused on water-soluble anti-oxidants (11, 12) rather than fat-soluble anti-oxidants. Therefore, the aim of the present study was to determine the effect of fat-soluble anti-oxidants in vegetable oils on acrylamide concentrations during the deep-fat frying of French fries. Three vegetable oils with different fat-soluble anti-oxidant contents were selected: i) commercial red palm oil, ii) yellow palm oil and iii) corn oil. Par-fried potato strips were fried in these oils, and the acrylamide concentrations in the French fries were measured at different times (frying temperature of 180 °C) and during 10 consecutive frying sessions. The fat-soluble anti-oxidant content and degradation of the quality of the oils were monitored before and after the 5th and 10th consecutive frying sessions.

## Materials and Methods

### Materials

This present study was conducted using shoestring par-fried potato strips that were 8 × 8 mm in cross section and 60–70 mm long. The potato strips was purchased from a hypermarket in Sri Serdang, Selangor, Malaysia. They were thawed for a minimum of 2 h at room temperature before frying. Three different vegetable oils with different fat-soluble anti-oxidant contents were used: i) commercial red palm oil, ii) yellow palm oil and iii) corn oil. The red palm oil commercially obtained from the market was a blend of 80% canola oil and 20% red palm oil (20). The vegetable oils were purchased from a hypermarket in Sri Serdang, Selangor. The par-fried potato strips were kept at −18 °C and the vegetable oils were kept at room temperature prior to use. Par-fried potato strips and vegetable oils from the same lot number were chosen.

### Frying Experiment

This study included two frying experiments. The effect of the frying conditions was determined in the first experiment, and the effect of consecutive frying was determined in the second experiment. In the first experiment, 250 g of potato strips was fried in an electric fryer (oil capacity: 5 L). Three different temperature and time combinations were used: (i) 180 ± 1 °C for 3.5 min to represent mild frying conditions, (ii) 180 ± 1 °C for 7 min to represent moderate frying conditions, and (iii) 180 ± 1 °C for 14 min to represent severe frying conditions. Napolitano et al. (12) studied the relationship between virgin olive oil phenol compounds and the formation of acrylamide in potato crisps and fried slices of potatoes at 180 °C for 5, 10, and 15 min. The three temperature and time combinations used by Napolitano et al. (12) was selected to be used in the present study with some modifications to suit the characteristics of materials used, i.e., par-fried potato strips. In the second experiment, 250 g of par-fried potato strips was fried in an electric fryer (oil capacity of 5 L) at 180 ± 1°C for 3.5 min. The frying started at approximately 15 min once the temperature of the oil reached 180 °C. Replicate frying experiments were conducted for each oil type. A new lot of oil was used for each replicate frying experiment. A total of ten consecutive frying sessions were conducted. In each consecutive frying session, the French fries were collected after the 1st, 5th and 10th frying. Samples were stored in clean plastic bags, labelled and kept at −18 °C before proceeding with the analysis. Approximately 100 mL of the vegetable oils was sampled before and after the 5th and 10th frying in each consecutive frying session. Oil samples were stored in amber glass bottles in a chiller (4 °C) prior to analysis. Replicate analyses of the peroxide value, *p*-anisidine value, iodine value, TOTOX value, free fatty acid content, fatty acid composition, and β-carotene, α-tocopherol and tocotrienol contents were performed on the collected oils.

### Analysis Methods

#### Materials

Acrylamide (> 99%) was obtained from Sigma–Aldrich (St. Louis, Missouri, USA). [^13^C_3_]-acrylamide (99% isotopic purity, 1000 mg kg^−1^) was obtained from Cambridge Isotope Laboratories, Inc. (Andover, Massachusetts, USA). Fatty acid methylesters (FAME) were obtained from Sigma–Aldrich (Bellefonte, PA, USA). Acetonitrile, hexane, formic acid, ammonium formate, and sodium methoxide were obtained from Merck (Darmstadt, Germany). The chemicals and solvents used were of analytical or high-performance liquid chromatography grade. Ultra-pure water was taken from an ELGA water purification system (Purelab Classic UV, Elga Labwater, Lane End, UK). The water was also used to prepare the stock solutions of acrylamide and ^13^C_3_-labeled acrylamide. The concentrations of the stock solution were 200 mg kg^−1^ and 4 mg kg^−1^, respectively. Working standards at concentrations of 1, 5, and 10 mg kg^−1^ were prepared by diluting the stock solution of acrylamide with water. The standard solution were stored in a chiller for a maximum of 3 months.

#### β-Carotene analysis

β-carotene was determined according to the method described by Kuntom (21). A sub-sample (0.10 g) of oil was weighed into a 25 mL volumetric flask. The oil samples were mixed and diluted with n-hexane until they reached the calibration mark. The sample was placed in a glass cuvette, and absorption was measured using a spectrophotometer (Genesys 20, 4001/4) at 446 nm.

### α-Tocopherol and Tocotrienol Analyses

#### Extraction

α-Tocopherol and tocotrienol concentrations were measured using a liquid chromatography tandem mass spectrometer (LC-MS/MS) according to Stephen (22). A subsample (0.03 g) of oil was weighed into a 10 mL amber vial, and 5 mL of n-hexane was added. The mixture was mixed using a vortex mixer (Model VRT-3000 L, LMS Co., Ltd., Tokyo, Japan) for 30 s and purged with nitrogen to dryness (Parker Domnick Hunter, Ohio, USA). Acetonitrile (5 mL) was added, and the solution was mixed using a vortex mixer for 30 s before it was immediately carried to a vial for the α-tocopherol and tocotrienol analyses.

#### UHPLC-MS/MS

The standards and extracts were injected into the Agilent 1290 Infinity ultra-high performance liquid chromatography (UHPLC) system via a Phenomenex Synergi Fusion RP column (2.1 × 100 mm, 3 μm) (Phenomenex, Torrance, CA, USA). The AB SCIEX QTRAP^®^ 5500 operating in the multiple reaction monitoring (MRM) mode was used to detect acrylamide. The Turbo VTM source was used with an electrospray ionisation (ESI) probe in positive polarity. The standards contained mixtures of tocopherol and tocotrienol at concentrations of 1, 3, 5, and 10 mg kg^−1^. The injection volume was set to 10 μL.

Separation of the tocopherol and tocotrienol components was achieved under gradient conditions using water with 0.1% formic acid and 5 mM ammonium formate (A) and acetonitrile with 0.1% formic acid and 5 mM ammonium formate (B) as the mobile phases at a flow rate of 500 μL/min. The gradient program was as follows: 0–2 min 70% A, 3–5 min. 0% A, 5–6 min 70% A. The run time for the sample extracts and calibration standards was 6 min.

#### Quantification

For quantification, the transitions *m/z* 431 > 165 for α-tocopherol, *m/z* 425 > 165 for α-tocotrienol, *m/z* 411 > 151 for γ-tocotrienol, and *m/z* 397 > 137 for δ-tocotrienol were used. The components of the vitamin E concentration in the samples were calculated based on the slope and the intercept value of calibration curve. The calibration curve was developed by plotting the peak area of each component of vitamin E against the concentration of the standards. The calibration curves were linear (*r*^2^ *>* 0.999). The limit of detection for α-tocopherol was 0.1 ng g^−1^, α-tocotrienol was 0.1 ng g^−1^, γ-tocotrienol was 0.2 ng g^−1^, and δ-tocotrienol was 0.5 ng g^−1^.

### Oil Qualities Analyses

AOCS official methods (23) were used to determine the peroxide value (Cd 8b-90), *p*-anisidine value (Cd 18–90), and free fatty acid content (Ca 5a-40) of the collected oil samples. The iodine value was determined using the MPOB Test Methods p 3.2. (21). The fatty acid composition was determined via transesterification based on the AOCS method No: Ce 1–62, 1998 (24). The oil was converted to fatty acid methyl esters (FAME) by dissolving 50 mg of oil in 950 μL of n-hexane, followed by the addition of 50 μL of sodium methoxide. The mixtures were vortexed (Uzusio VTX-3000 L, Tokyo, Japan) for 10 s and allowed to settle for 10 min at an ambient temperature of 25 °C. The top layer (1 μL) was injected into a gas chromatograph (Agilent 6890N, Little Falls, DE, USA) equipped with a split–splitless injector and a flame ionization detector. A medium polar cyanopropyl capillary column, DB23 (60 m × 0.25 mm × 0.15 μm) (Agilent Technologies, Santa Clara, CA, USA), was used. The FAME peaks were identified by comparing the retention times to a standard mixture. The peak areas were computed, and the percentages of the FAME were established as the area percentages via direct normalisation.

### Acrylamide Analysis

#### Extraction

The procedure was adapted from Sanny et al. (9) with some modifications. The French fries were ground in a blender (Braun multiquik ZK3, Frankfurt, Germany). A subsample (1.0 g) was put in a 50 mL centrifuge tube. Next, 10 mL of water containing the isotopically labelled acrylamide was added. The final concentration of the isotopically labelled acrylamide was 50 ng g^−1^. A vertical shaker (RS-1, Jeio Tech Co., Gyeonggi-do, Korea) was used to mix the solution for 30 min at ca 285 pulses/min. The homogenate was centrifuged in a centrifuge (3–18K, Sigma, Gillingham Dorset, UK) at a speed of 11,200 RCF (g) for 30 min at 4 °C. Approximately 2 mL of an aliquot beneath the oil layer was taken using a syringe and filtered through a 0.2 μm nylon syringe filter (Sartorius Stedim Biotech, Goettingen, Germany). The filtrate was immediately moved to a vial before proceeding with the acrylamide analysis.

#### UHPLC-MS/MS

The standards and extracts were injected into the Agilent 1290 Infinity UHPLC system via a Phenomenex Synergi Fusion RP column (2.1 × 100 mm, 3 μm) (Phenomenex, Torrance, CA, USA). The AB SCIEX QTRAP® 5500, which was operated in the MRM mode, was used to detect acrylamide. The Turbo VTM source was used with an ESI probe in positive polarity. The calibration standards comprised of acrylamide at five different levels: 1, 50, 250, 500, and 1000 ng g^−1^. ^13^C_3_-labeled acrylamide at a concentration of 50 ng g^−1^ was added to the calibration standards. The injection volume was set to 20 μL.

Acrylamide was separated under gradient conditions using water with 0.1% formic acid and 5 mM ammonium formate (A) and acetonitrile with 0.1% formic acid and 5 mM ammonium formate (B) as the mobile phases at a flow rate 250 μL/min. The gradient programme for acrylamide quantification using LC-MS/MS was 10% B to 90% B from 0.01 min to 4.0 min, hold for 1 min and back to 10% B in 0.1 min and reequilibration for 3 min. The run time for the analysis was 8 min.

#### Quantification

For quantification, the transitions *m/z* 72 > 55 for acrylamide and *m/z* 75 > 58 for ^13^C_3_-acrylamide were used. The transitions *m/z* 72 > 55, 72 > 54 and 72 > 44 were used to confirm the peak identity. A calibration graph was constructed by plotting the peak area of acrylamide relative to the internal standard against the corresponding ratios of the analyte amounts. The acrylamide concentrations in the sample extracts were calculated using the calibration slope and intercept value. The calibration curves were linear (r^2^ > 0.999). The limit of detection was 0.1 ng g^−1^.

### Statistical Analysis

The one-way ANOVA was used to determine the mean differences in the anti-oxidant contents and quality characteristics of the vegetable oils before and after the 5th and 10th consecutive frying sessions. Tukey’s multiple comparisons test was used to determine the significance of the differences in the anti-oxidant contents and quality characteristics of the oils. In addition, the two-way ANOVA was conducted to determine the difference in the mean of acrylamide between different types of vegetable oils and frying conditions as well as between different types of vegetable oils and number of frying. The significance of the differences in the acrylamide concentration was determined using Tukey’s multiple comparisons test. A *P*-value ≤ 0.05 in the tests was considered significant. Statistical analyses were performed using Minitab Statistical Software v.14 (Minitab Inc., State College, Pa., U.S.A.).

## Results and Discussion

The fat-soluble anti-oxidant contents in vegetable oils were determined to obtain insight into their initial levels and to monitor changes during 10 consecutive frying sessions. Table 1 indicates that the initial content of β-carotene was the highest in red palm oil (294 mg kg^−1^), which was followed by yellow palm oil (119 mg kg^−1^) and corn oil (84 mg kg^−1^). The results confirmed that red palm oil is the world’s richest food source of carotenoids (25). The initial content of α-tocopherol was the highest in corn oil (455 mg kg^−1^), and the initial total content of tocotrienol was the highest in yellow palm oil (425 mg kg^−1^). The content of α-tocopherol reported in the present study is consistent with those of Ramadan and Wahdan (26), who reported a similar content for α-tocopherol in corn oil at 417 mg kg^−1^. Further, Ryan et al. (27) confirmed that vegetable oils, such as corn oil, that are derived from grains and legumes are rich in tocopherol. Additionally, the total content of tocotrienol in yellow palm oil agrees with Adam et al. (28), who reported a similar total content of tocotrienol in yellow palm oil at 490 mg kg^−1^.

Moreover, Table 1 shows that the content of β-carotene, α-tocopherol and total tocotrienol decreased with the increasing number of frying for all types of oil. For instance, the content of β-carotene in yellow palm oil before frying significantly decreased from 119 mg kg^−1^ to 110 mg kg^−1^ after the 5th frying and further decreased to 82 mg kg^−1^ after the 10th frying session. The results are in agreement with those of Evuen et al. (29), who found that the β-carotene content in 10 different samples of vegetable oils was reduced significantly when the samples were subjected to repeated frying. The decrease in the α-tocopherol and total tocotrienol content with the number of frying in the present study is consistent with the results of other researchers (16) who found that various vitamin E fractions in palm oil (α-tocopherol, α-tocotrienol, γ-tocotrienol and δ-tocotrienol) were reduced when the oil was subjected to heat treatment. The findings indicated that the anti-oxidant contents could degrade to some extent due to their poor thermostability. Taghvaei and Jafari (30) highlighted that the poor thermostability of anti-oxidants at high heating temperatures or long heating times may reduce the opportunity to exert the anti-oxidant activity.

The quality characteristics of oil were determined to monitor the deterioration of oils during 10 consecutive frying sessions. Table 2 shows that the percentage of saturated and monounsaturated fatty acids, peroxide value, *p*-anisidine value, free fatty acid value, and TOTOX value increased with the number of frying for all oil types; however, the percentage of polyunsaturated fatty acid and the iodine value decreased. The results are in agreement with those of Lim et al. (10), who monitored the quality degradation in yellow palm oil during 10 consecutive frying sessions of sweet potato chips. They reported similar pattern of results; the percentage of saturated and monounsaturated fatty acids, peroxide value, *p*-anisidine value, free fatty acid value and TOTOX value increased, but the percentage of polyunsaturated fatty acid and the iodine value decreased with the increasing number of frying. The increase in the peroxide values implies that the amount of the primary oil oxidation products (such as hydroperoxides) increased, whereas the increased *p*-anisidine values indicate that the amount of secondary oil oxidation products (such as aldehydes and ketones) increased (31). The TOTOX value is a measure of the total oxidation, including primary and secondary oxidation products, and it gives a better estimation of the progressive, oxidative deterioration of fats and oils (31). Table 2 shows that the TOTOX value after the 10th frying session was the lowest in corn oil (29.7) followed by yellow palm oil (35.2) and red palm oil (38.5). In the present study, corn oil, which had the highest content of α-tocopherol, seems to be more effective in controlling oxidative deterioration during the frying of French fries than yellow and red palm oil. This result is in agreement with Alizadeh et al. (32), who studied the anti-oxidant activity of tocopherol on oil oxidation in a mixture of sunflower seed oil and palm olein during the deep frying of potato slices and reported that tocopherol is effective in controlling oil oxidation, especially during the primary stage. Although α-tocopherol seems effective for controlling oil oxidation, the effect of fat-soluble anti-oxidants in vegetable oils on acrylamide formation requires further research, as Constantinou and Koutsidis (15) indicated that different types of anti-oxidants with different structures exhibit varying degrees of carbonylbinding properties that inhibit acrylamide formation.

An experiment was conducted to establish the impact of the frying conditions on acrylamide using different vegetable oils. The frying temperature was held at 180 ± 1 °C, but the frying times were chosen to represent mild (3.5 min.), moderate (7 min.), and severe (14 min.) frying conditions. Table 3 shows that the acrylamide formation significantly increased with the increasing frying time (from the mild to moderate frying conditions) for red palm oil and corn oil, but not for yellow palm oil. Acrylamide formation slightly dropped under the severe frying condition all types of oil, but the difference was not significant. The acrylamide concentration in the French fries prepared using red palm oil significantly increased from 740 ng g^−1^ under the mild condition to 852 ng g^−1^ under the moderate condition and slightly dropped to 828 n/g under the severe condition. The increase in the acrylamide concentration from the mild to moderate frying conditions reported in the present study is in agreement with the results of Napolitano et al. (12), who reported that the acrylamide concentration of potato crisps rapidly increased with the increasing frying time at the same temperature (180 °C). Further, the decrease in the acrylamide concentration from the moderate to severe condition reported in the present study is consistent with the findings of Rydberg et al. (33), who reported that prolonged heating may decrease acrylamide formation as a result of a degradation process and exhaustion of one of the reactants. The results in the present study confirm the findings of other researchers. Knol et al. (34) reported that acrylamide is subjected to complex reactions (formation as well as degradation) because acrylamide is an intermediate product of the Maillard reaction.

Table 3 shows no significant differences in the acrylamide concentrations in the French fries fried under moderate and severe frying conditions for all types of oil. The effect of the fat-soluble anti-oxidants in vegetable oils to reduce acrylamide formation under different frying conditions was not evident in this experiment. To observe the effect of fat-soluble anti-oxidants in vegetable oil on acrylamide formation, a much longer frying time was used, and consecutive frying sessions were conducted. Table 4 shows that no significant difference in the acrylamide concentration in French fries was observed with the increasing number of frying in yellow palm oil. However, the acrylamide concentration significantly dropped to the lowest level, at 524 ng g^−1^, after the 10th frying session in red palm oil. These results are in contrast to those of Urbančič et al. (35), who studied the effects of rosemary extract on sunflower oil stabilisation and found that acrylamide formation increased during 20 deep frying sessions of potatoes. Our findings are in contrast with the results of previous research possibly due to the decomposition of rosemary and other water-soluble anti-oxidants during deep frying (36). Kamaruzaman et al. (37) reported that red palm oil has a high oxidative stability and contains high levels of fat-soluble anti-oxidants, such as vitamin E and carotenoids. The content of β-carotene after the 10th frying session was the highest in the red palm oil (143 mg kg^−1^). Further, Table 4 shows that the acrylamide concentration in French fries after the 10th frying session was significantly lower using red palm oil (524 ng g^−1^) than yellow palm oil (779 ng g^−1^) and corn oil (813 ng g^−1^). As mentioned, the Maillard reaction is the major mechanistic pathway of acrylamide formation (6). Carbonyl scavenging has been proposed to effectively reduce or prevent acrylamide formation by interrupting critical steps or scavenging key intermediates in the acrylamide formation pathways (38). Since the Maillard reaction and mechanisms for the formation of acrylamide may involve free radicals, it has been proposed that anti-oxidants may scavenge these free radicals and decrease the formation of acrylamide (39). Possibly, β-carotene in red palm oil may scavenge free radicals and decrease the formation of acrylamide during the frying of French fries. We reported earlier that the type of vegetable oil signiﬁcantly inﬂuenced acrylamide formation in sweet potato chips (10). In the present study, we report that fat-soluble anti-oxidants in vegetable oils do contribute to signiﬁcant differences in acrylamide concentrations during the deep-fat frying of French fries. Using red palm oil, which has a high content of β-carotene, signiﬁcant changes in the acrylamide concentration were seen. Particularly, acrylamide formation slowed down when red palm oil, which is rich in β-carotene, was used. However, the effect of fat-soluble anti-oxidants in red palm oil on the formation of acrylamide was more apparent during consecutive frying than different frying conditions. The overall results of this study suggest that the use of red palm oil for deep-fat frying French fries can be used as a mitigation strategy to reduce acrylamide formation, but further studies are necessary to investigate the influence of different types of fat-soluble anti-oxidants on inhibiting acrylamide formation.

## Conclusion

The effect of fat-soluble anti-oxidants in red palm oil to reduce acrylamide formation in French fries was more apparent when a prolonged frying time was used under consecutive frying sessions than under different frying conditions. The content of β-carotene after the 10th frying session was the highest in the red palm oil. It was proposed that β-carotene in the red palm oil may scavenge key intermediates in the acrylamide formation pathways and decrease the formation of acrylamide during the frying of French fries. Deep-fat frying is commonly practiced in the food industry, food service establishments as well as homes; the use of red palm oil can be anticipated as a mitigation strategy to reduce acrylamide formation in deep-fried, carbohydrate-rich foods. In addition, we showed that corn oil had the highest content of α-tocopherol and was somewhat more effective in controlling oxidative deterioration during the frying of French fries than yellow and red palm oil. Although α-tocopherol seems to be effective in controlling oil oxidation, the effect of fat-soluble anti-oxidants in vegetable oils on acrylamide requires further research, as different types of anti-oxidants with different structures exhibit varying degrees of carbonyl-binding properties for inhibiting acrylamide formation.

## Figures and Tables

**Table 1:** Antioxidants content of different vegetable oils before, after 5th and 10th consecutive frying sessions

Antioxidants content, mg kg^−1^	Types of vegetable oil								

	Red palm oil			Yellow palm oil			Corn oil		
		
	Before frying	After 5th frying	After 10th frying	Before frying	After 5th frying	After 10th frying	Before frying	After 5th frying	After 10th frying
β-carotene	294^a^(0.099)	196^b^(0.184)	143^c^(0.127)	119a′(0.134)	110b′(0.071)	82c′(0.721)	84a″(0.672)	79b″(0.113)	77c″(0.70)
α-tocopherol	197^a^(43.8)	130^a^(29.3)	91^a^(12.3)	42a′(4.58)	34a′(3.18)	19b′(1.01)	455a″(20.8)	392b″(1.30)	413^a^b″(14.3)
α-tocotrienol	36^a^(4.49)	34^a^(3.16)	32^a^(0.11)	114a′(39.5)	89a′(26.7)	45b′(13.4)	24a″(9.26)	20a″(4.44)	21a″(1.89)
γ-tocotrienol	75.8^a^(17.2)	68.8^a^(8.45)	62.5^a^(1.39)	249a′(43.5)	235a′(40.6)	148b′(27.5)	14.2a″(0.24)	19.9a″(7.19)	26.8a″(4.80)
δ-tocotrienol	12.6^a^(1.93)	13.4^a^(0.61)	12.5^a^(1.66)	62.4a′(7.89)	36.2b′(3.76)	23.4c′(2.03)	0.64a″(0.08)	BDL	BDL
Total tocotrienol	124^a^(0.63)	116^b^(1.84)	107^c^(1.05)	425a′(22.1)	360a′(21)	216b′(9.76)	38.6a″(0.45)	39.6a″(0.82)	47.6a″(1.02)

a–c Values within the same row with different letters among different treatments using red palm oil are significantly different (*P* ≤ 0.05)

a′–c′ Values within the same row with different letters among different treatments using yellow palm oil are significantly different (*P* ≤ 0.05)

a″–c″ Values within the same row with different letters among different treatments using corn oil are significantly different (*P* ≤ 0.05)

Values are means of duplicate determination (±SD)

BDL = below detection limit

**Table 2:** Quality characteristics of different vegetable oils before, after 5th and 10th consecutive frying sessions

	Types of vegetable oil								

	Red palm oil			Yellow palm oil			Corn oil		
		
	Before frying	After 5th frying	After 10th frying	Before frying	After 5th frying	After 10th frying	Before frying	After 5th frying	After 10th frying
Peroxide value, meq kg^−1^ oil	7.12^a^(0.29)	12.5^b^(0.19)	12.9^b^(0.46)	7.09a′(0.22)	12.4b′(1.16)	13b′(0.52)	8.81a″(0.13)	9.13a″(0.04)	9.53b″(0.07)
Iodine value, g of I 100 g^−1^ oil	92.9^a^(1.43)	88.2^b^(0.59)	83.7^b^(1.05)	64.8a′(0.08)	56.9b′(0.07)	54.5c′(0.42)	93.3a″(0.09)	92.8a″(0.18)	85.3b″(0.91)
*p*-Anisidine value	6.61^a^(0.33)	10.7^b^(0.32)	12.8^c^(0.16)	2.33a′(0.06)	4.17b′(0.13)	9.19c′(0.13)	3.92a″(0.07)	6.42b″(0.09)	10.7c″(0.11)
Free fatty acids, %	0.09^a^(0.014)	0.15^b^(0.006)	0.21^c^(0.006)	0.09a′(0.003)	0.11a′(0.003)	0.11a′(0.004)	0.12a″(0.014)	0.13a″(0.014)	0.13a″(0.021)
TOTOX value, 2 PV+AV	20.6^a^(1.97)	35.5^b^(1.55)	38.5^c^(0.54)	16.5a′(3.04)	28.9b′(7.89)	35.2c′(0.98)	21.8a″(4.56)	24.7b″(0.66)	29.7b″(3.63)
Fatty acid distribution, %									
Saturated fatty acid	48.0^a^ (0.47)	49.5^a^(0.51)	51.9^b^(0.26)	38.6a′(0.38)	40b′(0.07)	41.2c′(0.05)	14.3a″(0.04)	14.8b″(0.09)	15.2c″(0.15)
Monounsaturated fatty acid	33.5^a^(0.12)	34^b^(0.01)	34.3^c^(0.05)	35.1a′(0.13)	35.5a′(0.42)	35.9a′(0.07)	10.9a″(0.03)	11.7b″(0.10)	19c″(0.10)
Polyunsaturated fatty acid	19.9^a^(0.06)	16.6^b^(0.42)	13.5^c^(0.70)	25.4a′(0.78)	23.4^a^b′(0.18)	21.4b′(0.11)	74.1a″(0.30)	73.2a″(0.15)	66.6b″(0.33)

a–c Values within the same row with different letters among different treatments using red palm oil are significantly different (*P* ≤ 0.05)

a′–c′ Values within the same row with different letters among different treatments using yellow palm oil are significantly different (*P* ≤ 0.05)

a″–c″ Values within the same row with different letters among different treatments using corn oil are significantly different (*P* ≤ 0.05)

Values are means of duplicate determination (±SD)

**Table 3:** Acrylamide concentration of French fries at 180 °C using different vegetable oils at different frying conditions

Frying condition	Types of vegetable oil		

	Red palm oil (ng g^−1^)	Yellow palm oil (ng g^−1^)	Corn oil (ng g^−1^)
Mild	740 aB (1.41)	777 aA (37.48)	736 aB (8.49)
Moderate	852 aA (11.31)	789 bA(3.54)	804^b^^A^ (6.36)
Severe	828 aA (14.14)	753 aA (41.01)	801^a^^A^ (2.12)

a–b Values within the same row with different letters are significantly different (*P* ≤ 0.05)

A–B Values within the same column with different letters are significantly different (*P* ≤ 0.05)

Values are means of duplicate determination (±SD)

**Table 4:** Acrylamide concentration of French fries (frying at 180 ± 1 °C for 3.5 min) using different vegetable oils after 1st, 5th and 10th consecutive frying sessions

Number of frying	Types of vegetable oil		

	Red palm oil (ng g^−1^)	Yellow palm oil (ng g^−1^)	Corn oil (ng g^−1^)
After 1st frying	853 aA (38.89)	840 aA (35.36)	810 aB (1.41)
After 5th frying	848 aA (31.11)	776 aA (11.31)	824 aA (2.83)
After 10th frying	524 bB (89.80)	779 aA (2.12)	810 aB (4.24)

a–b Values within the same row with different letters are significantly different (*P* ≤ 0.05)

A–B Values within the same column with different letters are significantly different (*P* ≤ 0.05)

Values are means of duplicate determination (±SD)
